# Influence of Cooking Oil on the Mitigation of Autogenous Shrinkage of Alkali-Activated Slag Concrete

**DOI:** 10.3390/ma13214907

**Published:** 2020-10-31

**Authors:** Jinguang Huang, Jiachuan Yan, Kaihua Liu, Bin Wei, Chaoying Zou

**Affiliations:** 1 School of Civil Engineering, Harbin Institute of Technology, 73 Huanghe Rd, Nangang, Harbin 150090, China; huangjinguang@hit.edu.cn (J.H.); kaihualiu@hit.edu.cn (K.L.); dragon6693@163.com (B.W.); 2 Key Lab of Structures Dynamic Behavior and Control of the Ministry of Education, Harbin Institute of Technology, 92 Xidazhi St, Nangang, Harbin 150090, China; 3 Key Lab of Smart Prevention and Mitigation of Civil Engineering Disasters of the Ministry of Industry and Information Technology, Harbin Institute of Technology, 92 Xidazhi St, Nangang, Harbin 150090, China

**Keywords:** emulsified cooking oil, autogenous shrinkage, alkali-activated slag concrete, durability

## Abstract

This paper reports an investigation into the autogenous shrinkage, mechanical, and durability performances of alkali-activated slag concrete (AASC) with emulsified cooking oil (ECO). Properties of AASC including flowability, setting time, compressive strength, autogenous shrinkage, and carbonation depth are tested to clarify the effects of the ECO. Commercially available expansion agent (EA) and shrinkage reducing agent (SRA) are also applied on AASC to compare with ECO. Experimental results show that the utilization of ECO could significantly decrease the autogenous shrinkage of alkali-activated slag concrete owing to the reduction of surface tension and the denser internal structure. It also shows that cooking oil after emulsification could have better performances than that of plain cooking oil when applied on AASC. Setting time and carbonation resistance ability are also improved with the utilization of ECO. The application of ECO is considered a cheap and easy way to overcome the limitation of AASC.

## 1. Introduction

Production of ordinary Portland cement is currently a serious source of global warming. Roughly 0.7–1 ton of CO_2_ is released while producing 1 ton of cement [1]. About 7% of global CO_2_ emissions is contributed by cement manufacture-related activities, and the production of cement is considered as the third largest producer of greenhouse gas [2,3].

Therefore, numerous studies have been focusing on eco-friendly alternative supplementary cementitious materials to limit the emission of CO_2_. Ground granulated blast furnace slag (GGBFS) is a type of industrial by-product obtained during pig-iron manufacturing and is generally used as supplementary cementitious materials [4]. Compared with ordinary Portland cement (OPC)-based binder, the application of GGBFS can remarkably decrease hydration heat and increase the durability of concrete [5]. However, the relatively low early age strength of GGBFS limits its applications [6]. Since the 1940s, researchers have found that alkali activation could overcome the shortage of low early age strength of GGBFS-contained binders [7,8]. A series of investigations into alkali-activated slag binder have been carried out in the past two decades [9,10,11,12,13]. The utilization of alkali-activated slag binder or alkali-activated slag concrete (AASC) can provide superior performances including superior mechanical performance, higher resistance to chemical attack, frost-thaw performance, lower heat of hydration, lower permeability, and good thermal performance [14,15,16,17].

Despite environmental and property advantages, one problem limiting the application of AASC is high shrinkage, which is one of the main factors contributing to early age cracking [18]. Shrinkage in concrete mainly has two forms: Drying shrinkage and autogenous shrinkage. In AASC, which form of shrinkage being dominant is still controversial [19]. The shrinkage in AASC is reported about 1.7–2 times larger than the OPC-based concrete [20,21,22]. High shrinkage may cause cracks and degrade the durability of AASC. The service life of AASC may be highly shortened due to high shrinkage and the maintenance costs of repairing cracks may also dramatically increase.

To solve the shrinkage problem, many researchers are focused on the utilization of admixtures to mitigate the shrinkage of AASC. Expansive agents (EA) and shrinkage reducing agents (SRA) are two types of widely used admixtures to inhibit concrete shrinkage [22,23,24]. However, most of the EA and SRA are based on OPC concrete and not all admixtures work effectively in AASC. For instance, gypsum is one type of commonly used EA in OPC-based concrete, but the application of gypsum in AASC is reported insufficient to compensate for long age shrinkage [22]. Moreover, the adding amount of EA is difficult to quantify and several negative side effects on the properties of AASC may also be generated [22,24,25]. Other research have focused on using lightweight aggregates and super absorbent polymers as internal curing materials in AASC or using an elevate temperature curing method to mitigate shrinkage [26]. These approaches have had a good effect on the mitigation of shrinkage in AASC. However, these methods are considered high cost approaches in a practical application point of view and some of these approaches are also considered less suitable to use in-situ. Therefore, a new strategy to mitigate the shrinkage of AASC with high added value and applicability to in-situ is still needed.

Cooking oil is a type of necessity which could be easily obtained around the world. Recently, some studies focused on the application of cooking oil on concrete [27,28,29,30,31,32,33]. It has been reported that after being pretreated by cooking oil, recycled aggregate has shown better performance in asphalt concrete [32]. The utilization of linseed oil could enhance the durability of lime-based mortar [29]. A few studies have also used cooking oil as a shrinkage-reducing agent in OPC-based concrete [27]. Shrinkage of OPC-based concrete is significantly reduced by the utilization of oil-based admixture due to the reaction between CaO and fatty acid [27]. GGBFS also contains a large quantity of CaO, and the chemical reaction between CaO and fatty acid may also be generated, therefore the addition of cooking oil may theoretically decrease the autogenous shrinkage in AASC. In addition, some researchers have found that shrinkage could be reduced significantly by decreasing the surface tension of the pore solution in concrete [34]. As known, the surface tension of the solution highly depends on its density and cooking oil has a lower density than water. Thus, the surface tension of the water/oil solution after emulsification is lower than pure water, and the lower surface tension by applying cooking oil in AASC is possibly another factor that is predicted to decrease the shrinkage of AASC. In comparison to OPC-based concrete, the autogenous shrinkage problem of AASC is even more serious, and as known, no studies have focused on this aspect. Therefore, the potential application of cooking oil as a shrinkage reducing agent into AASC is necessary.

The primary aim of this article is to investigate the effects of plain cooking oil and emulsified cooking oil (ECO) on the shrinkage-reducing performance of AASC. Commercially available EA and SRA are also applied to AASC for comparison with cooking oils. First, experiments on paste are carried out to compare the effects of flow and setting time of the alkali-activated slag paste with four different agents. Then, four mixing approaches, each with the same dosage of four kinds of agents, are explored to evaluate the effect of cooking oil on the durability and mechanical properties of AASC. Compressive strength, autogenous shrinkage, and carbonation depth are tested. Finally, surface tension experiment, mercury intrusion porosimetry (MIP) and SEM analysis are also carried out to discuss the mechanism of the shrinkage-reducing effect of ECO.

## 2. Materials and Methods

### 2.1. Materials

#### 2.1.1. Alkali Activator (GGBFS)

**Figure 1:**
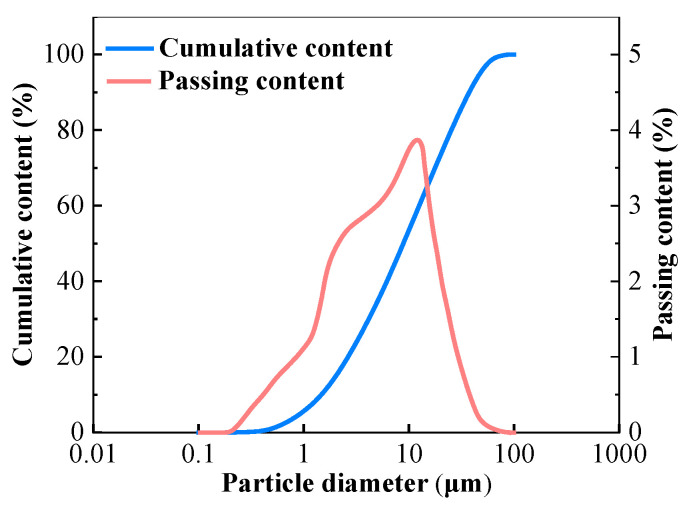
Particle size distribution of GGBFS.

The GGBFS used as the primary raw material was produced in Shijiazhuang, China with a density of 2940 kg/m^3^ and surface area of 410 m^2^/kg. The basicity coefficient (the ratio of (CaO + MgO)/(Al_2_O_3_ + SiO_2_)) and quality coefficient, (CaO + MgO + Al_2_O_3_)/(SiO_2_ + TiO_2_), according to the chemical composition were 1.09 and 1.98, respectively. The chemical composition of GGBFS determined by X-ray fluorescence (XRF) is listed in Table 1. Figure 1 shows the size distribution of particles of GGBFS analyzed by a Laser Particle Size Analyzer provided by Shengzhong Instrument company (Qingdao, China).

#### 2.1.2. Alkali Activator

In this study, water glass and sodium hydroxide were used as the alkali activator. Sodium hydroxide was mixed in the commercially available water glass solution to adjust the Ms (Ms, SiO_2_/Na_2_O) of the solution so as to be equal to one. The commercially available water glass provided by Yousuo Chemical Industry (Shijiazhuang, China) with 27.06 wt.% SiO_2_ and 8.57 wt.% Na_2_O was used. The sodium hydroxide with the purity of 99% used to adjust the Ms was provided by a Hengxing Chemical Industry (Tianjin, China).

#### 2.1.3. Aggregates

River sand with a maximum particle size of 4.75 mm and crushed gravel with a maximum particle size of 20 mm were used as fine and coarse aggregates, respectively. Both the fine and coarse aggregates were obtained from local sources (Harbin, China). The properties of river sand and crushed gravel are listed in Table 2.

#### 2.1.4. Agents

Commercially provided EA based on sulphoaluminate and SRA based on a polyether derivative were used to compare with cooking oil and ECO. Span-80 and OP-10 emulsifiers were a collocation used as the emulsification agents. The properties of these agents are summarized in Table 3. Commercially available vegetable oil with a density of 0.88 g/cm^3^ was used as the cooking oil.

### 2.2. Details of Mixing Proportions and Casting of Specimens

#### 2.2.1. Emulsification of Cooking Oil

The stability of the emulsion is important for applying ECO. Before adding ECO to AASC, different types and dosages of the emulsification agents were examined. The components of emulsified water/cooking oil solution used in this study are shown in Table 4, which shows a superior stability than other tested emulsions.

The preparation of the emulsification of the cooking oil is listed as followed:

- All cooking oil was poured into a conical flask and warmed to 60 °C utilizing a thermostat water bath, and mixed at the speed of 1200 r/min for 30 min;
- A total of 66.7% of the total water and all of the emulsifying agents were added into the conical flask with the same mixing speed for 20 min;
- Finally, the rest water was poured into the solution with the same mixing speed for another 20 min.

**Figure 2:**
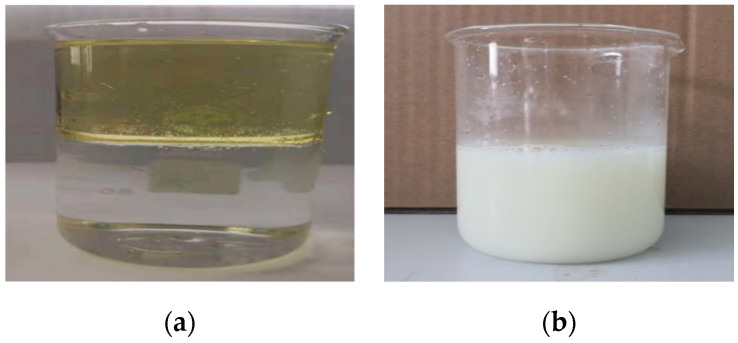
Water and oil solution (**a**) before and (**b**) after emulsification.

The difference between the cooking oil/water solution before and after emulsification could be observed in Figure 2.

#### 2.2.2. Mixing of Paste

The paste with 3 mass% of Na ion by adding alkali activator into GGBFS was produced for flow, setting time, and MIP tests. The tests for flow and setting time were with the agents dosage of 2 mass% of GGBFS. For the MIP test, pastes with ECO of 0 mass%, 2 mass%, and 4 mass% of GGBFS were carried out.

#### 2.2.3. Mixing of Concrete

The water to binder ratio (W/B) of the concrete was fixed at 0.5. This level of W/B is considered to be commonly used and of acceptable workability, and the compressive strength is predicted to be higher than 50 MPa to obtain high enough autogenous shrinkage in AASC so as to enable a direct comparison in different approaches. The unit water volume and fine aggregate ratio (S/a) of the AASCs were fixed at 180 kg/m^3^ and 40%, respectively. By using this mixture proportion, good workability and mechanical property could be obtained. For the alkali activator, the concentration of Na ion was fixed at 3 mass% of GGBFS. The dosages of four kinds of agents were 2 mass% of GGBFS. The concrete mixture proportions are shown in Table 5. Aggregates and GGBFS were dry mixed for 1 min, and NaOH, water glass, and part of the free water were pre-mixed with water. After dry mixing, the alkali activator and rest free water were added into the concrete and mixed for 90 s. Then, four kinds of agents were added and mixed for 1 min. The water contained in the ECO and alkali activator was removed from free water.

### 2.3. Test Methods

#### 2.3.1. Test for Alkali-Activated Slag Paste (AASP)

The flow of the paste with different agents were tested according to GB/T 8077-2000 [35]. A total of 300 g of binder and 105 g of water were mixed and filled in a Vicat ring on a glass plate. The tip of the plunger was felled onto the top surface of the paste and then set free for more than 30 s. The average value of the maximum diameter of two directions perpendicular to each other was regarded as the result of the paste flow.

The initial and final setting times of paste with various agents were measured by using the Vicat apparatus provided by Meiyu Instrument company (Shanghai, China) in accordance with the ASTM C191 [36]. The paste was filled into the Vicat ring on the glass plate, the needle of the Vicat was placed onto the top surface of the cement paste and tightened with a screw. Upon loosening the screw, the needle was plunged in different points of the paste every 5 min by changing the position of the ring. When the needle plunging into the paste fell to a distance of 3–5 mm from the glass plate, it was regarded as the initial setting time. After the initial setting time, the needle was plunged every 15 min. The final setting was determined when the needle could be plunged 1 mm below the surface of the paste.

The distribution of the pore size was determined by utilizing mercury intrusion porosimetry (MIP) test. An AutoPore IV-9500 V 1.09 type mercury porosimeter with a maximum pressure of 3300 psia was used for the MIP test. Approximately 10 g of each sample was core drilled from a 100 mm × 100 mm × 100 mm cubic specimen after curing at 20 ± 2 °C and 95% RH environment for 28 days. The cored samples were dried for approximately 24 h at 60 °C till a constant weight was reached, and then immersed in mercury under gradually increased pressure within a fully enclosed pressure chamber. With the increase of pressure, the mercury forced into the pores of the samples, the total porosities and radius of pores of the samples could be determined by this test. It should be noted that there are some limitations of MIP technique [37,38,39], such as the possibility of losing hydrated water from the reaction products and by oven drying. Utilizing a vacuum drying method could be a better choice than drying at 60 °C [40].

Backscattered electrons (BSE) mode was used to measure the micro-cracks of the specimens. For SEM analysis, samples were prepared by crushing the pastes into small pieces with dimensions of approximately 2 cm^3^ from the specimens after curing at 20 ± 2 °C and 95% RH environment for 28 days. The slices were immersed in pure ethyl alcohol for 24 h to stop the hydration of the binders. For the observation of BSE imagines, samples were impregnated using a low viscosity epoxy resin and polished down to ¼ μm. Before observation, conductive coating was applied to the polished sections of the samples.

The surface tension of the ECO solution was tested by using the BZY surface tensiometer provided by Fangrui Instrument Company (Shanghai, China). After being preheated for 30 min, the platinum plate was washed by running water. Then, the sample was placed in the sample stage. The knob was controlled to raise the sample stage until the liquid level of the sample contacted with the platinum. The digit on the display screen was slowly raised due to the surface tension of the sample. Finally, a readout of the digit on the display screen was done as the final result of the surface tension when the digit was constant.

#### 2.3.2. Test for AASC

The compressive strength test was in accordance with GB/T 50081-2019 [41]. In totally, 100 mm × 100 mm × 100 mm cubic specimens were used for the compressive strength test at the 7th and 28th day. Specimens were de-moulded at one day after pouring, then cured in a curing room with RH of 95% and temperature 20 ± 2 °C. Each measured value is an average of three measurements.

The carbonation test strictly followed GB/T 50082-2009 [42]. The 100 mm × 100 mm × 400 mm specimens were used for the carbonation test. After pouring, specimens were cured in a curing room with RH of 95% and temperature 20 ± 2 °C. Surfaces except the exposed two surfaces were sealed with heated paraffin wax. Then, specimens were exposed in the carbonation chamber at 20 ± 2 °C and 70 ± 5% RH. Carbonation depth was tested at the 3rd, 7th, 14th, and 28th days after being exposed in the carbonation chamber. A total of 1% phenolphthalein solution in alcohol was sprayed on a fracture surface of the concrete and the depth of neutralization was measured. Average values of three specimens were used as the final result for each testing age.

**Figure 3:**
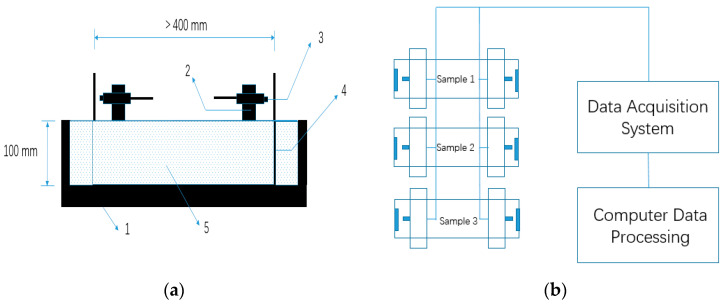
Autogenous shrinkage test method for alkali-activated slag concrete (AASC): (**a**) Non-contact concrete shrinkage deformation test system. 1. Steel mold; 2. Sensor support; 3. Eddy-current displacement sensor (ECDS) 4. Standard target; 5. Concrete mixture, and (**b**) Schematic diagram of autogenous shrinkage measurement for AASC.

The autogenous shrinkage test was also in accordance with GB/T 50082-2009 [42]. The 100 mm × 100 mm × 515 mm specimens were used for the autogenous shrinkage test. A SME-NCC-2 type eddy-current displacement sensor (ECDS) based on the electromagnetic induction effect was used to measure the autogenous shrinkage. Compared with generally used non-contact sensors such as a laser-based sensor and capacitive sensors, measurement by ECDS has advantages of tolerance to a dirty environment and is not sensitive to the material in the gap between sensor and target [43]. The details of the experimental method were identical to those described in the publications of other authors [43,44]. Mixtures were cast into the steel mould immediately after mixing, and steel target seats were embedded into specimens to make the deformation simultaneously with AASCs. The surface of the mixtures was sealed with two layers of polyethylene sheets to insulate from exterior drying. The exterior temperature was sustained at 20 ± 2 °C. The test of autogenous shrinkage in this study lasted for seven days after pouring. The geometry of the samples for the measurement of autogenous shrinkage is shown in Figure 3.

## 3. Results and Discussion

### 3.1. Flow of the AASP

**Figure 4:**
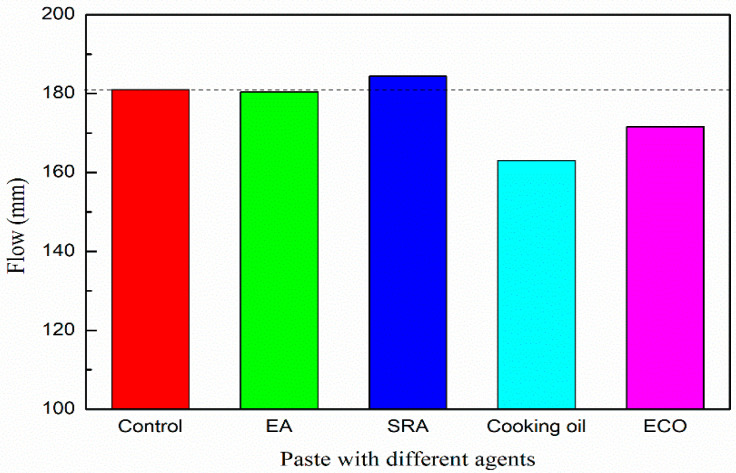
Flow of paste with various of agents.

Figure 4 shows the flow values of paste with various types of agents. All the dosages of the agents were fixed at 2 mass% of GGBFS. The flow of the paste without agent is 181.0 mm. Flow values of pastes with EA and SRA are 180.4 mm and 184.4 mm, respectively. The addition of EA and SRA shows very limited influence on the flow value of the alkali-activated paste. With the addition of cooking oil, the flow value decreases to 163.0 mm, which is approximately 10% lower than plain. It may be due to the cooking oil itself being more viscous than pure water, and therefore the addition of cooking oil increases the viscosity of the paste. The flow of the paste with ECO increases to 171.6 mm, which is approximately 5% higher than the mixture with the cooking oil owing to the homodisperse attributed to the emulsification. After emulsification, an excellent dispersion of cooking oil can be realized in the paste, oil droplets dissolve in the mixture and decrease the viscosity, and the increased dispersion leads to a decrease of viscosity, thus increasing the flow with respect to the mixture with the cooking oil. Even for the lowest flow value (mixture with cooking oil), the decrease range of flow value is within 10% with respect to the plain mixture and is considered to be in acceptable range.

### 3.2. Setting Time

The setting times of the pastes with different agents are shown in Table 6. Generally, the mixtures show similar final setting times regardless of the types of agents except the EA-contained mixture. However, the initial setting times are sensitively dependent on the types of agents.

The initial and final setting times of the control mixture without any agent are 46 min and 123 min, respectively. The setting of paste becomes quicker when EA is added. This could be explained by the rapid hardening property of sulphoaluminate contained in EA [45,46]. Setting time is prolonged by the addition of SRA. This result complies with other researches [47,48] and the addition of SRA may modify the morphology of the hardened paste. Moreover, SRA affects the formation of hydration products and delays the setting of the binder.

With the addition of cooking oil and ECO, the initial and final setting times are prolonged. According to other research, hydration may be delayed due to a covering insolubility film on the GGBFS particles caused by the addition of cooking oil, hence increasing the setting time of AASC [28]. Another possible reason for the increase of the setting time may be attributed to the saponification reaction between CaO and fatty acid. The fatty acid may be adsorbed to Ca(OH)_2_ particles, forming micelles consisting of fatty acid ions and calcium salts of fatty acids, which may hinder the early hydration reaction among binder particles [49].

The utilization of ECO can retard the initial and final setting times by 108.6% and 21.1%, respectively, compared with the control mixture. The rapid setting is one of the problems in the application of AASC in-situ [50]. Especially for the high strength AASC, the initial setting of AASCs is extremely fast, resulting in a short duration of plastic condition for AASC to be used in-situ. The utilization of ECO can obviously retard the initial setting of AASC and may be a solution to resolving the rapid setting of AASC.

### 3.3. Compressive Strength of the Concrete

**Figure 5:**
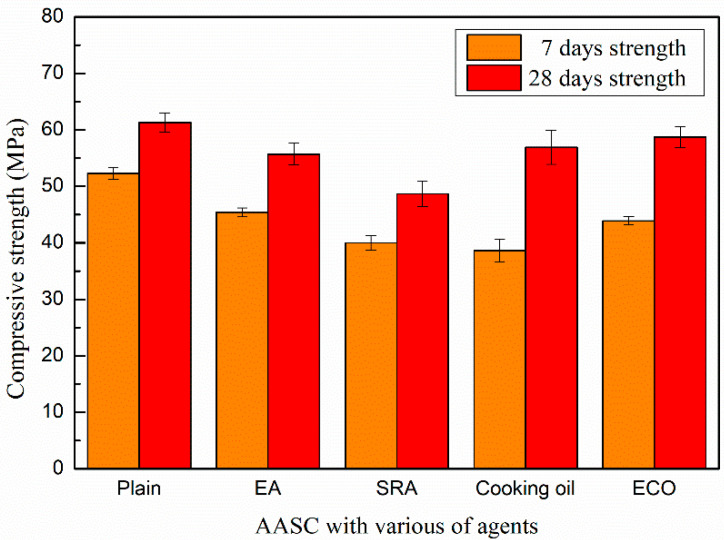
Compressive strength of AASC with various of agents.

The compressive strengths of AASCs with different agents are shown in Figure 5. The addition of agents considered here reduces the compressive strength, most prominently at the early age.

The compressive strength of the control mixture without agents on the 7th day is 52.3 MPa, while the strengths of specimens with EA and SRA are 45.4 and 40.0 MPa, respectively. At the 28th day, the compressive strengths of specimens with EA and SRA are 55.7 and 48.7 MPa, respectively, which are decreased by 9.1% and 20.5% in comparison with that of the control AASC after a same age time.

The addition, EA results in a significantly increase of the volume of the concrete, due to the formation of secondary ettringite, may lead to a possible micro-crack and higher porosity in concrete [48,51]. Thus, the compressive strength is decreased by the application of EA.

The decreased strength results by applying SRA are mainly attributed to its setting and hydration retarder property. The addition of SRA modifies the morphology and shows a negative influence on strength development [48,51].

With the addition of the cooking oil and ECO, the compressive strengths sharply reduce to 38.6 MPa and 43.9 MPa on the 7th day. The loss of early age strengths caused by the addition of cooking oil and ECO is generally due to the delayed hydration by a covering insolubility film on the GGBFS particles [28]. Moreover, another reason for the loss of early age strength may be owing to the saponification reaction between fatty acid in cooking oil and CaO. The fatty acid may be adsorbed to Ca(OH)_2_ particles and form micelles consisting of fatty acid ions and calcium salts of fatty acids, which may hinder the early hydration reaction among binder particles, thus decreasing early age strength [49].

For strength at the 28th day, specimens with the addition of ECO are similar with the control mixture. It is considered that the restraining of the hydration caused by ECO may become weaker with developing age. ECO may also lead to the refinement of the pores and decrease the porosity in AASC, as will be illustrated by the MIP data in Section 3.6.1. This may somehow increase the long age strength of AASC.

Specimens with cooking oil show lower strength than those with ECO on the 28th day. As known, cooking oil is not dissoluble in water and the un-reacted cooking oil may lead to defects in the AASC and cause loss of compressive strength.

### 3.4. Autogenous Shrinkage of the Concrete

**Figure 6:**
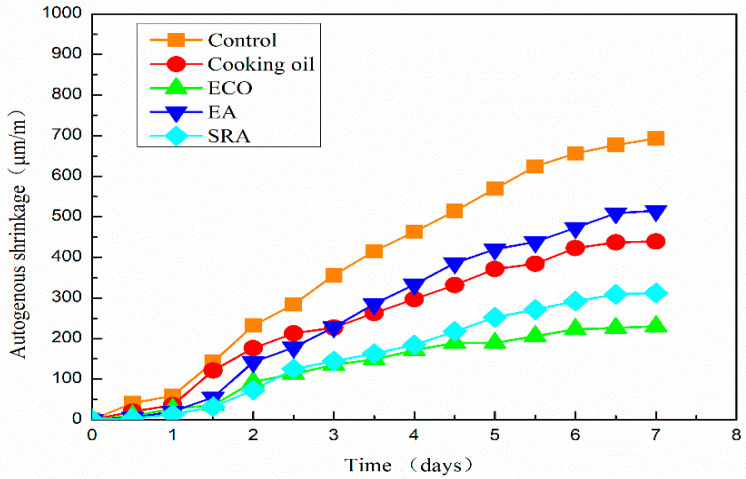
Autogenous shrinkage of AASC with various of agents.

The autogenous shrinkage results of all AASC specimens are shown in Figure 6. It shows that the shrinkage of the specimen without any agents is 693 μm/m on the 7th day. Autogenous shrinkage is significantly reduced with the addition of all kinds of agents. On the 7th day, the autogenous shrinkages of specimens with cooking oil, ECO, EA, and SRA are 439 μm/m, 230 μm/m, 515 μm/m, and 312 μm/m, respectively, lower than the control specimen without agents. Autogenous shrinkages are sharply reduced by 25.7% and 66.8% by the application of cooking oil and ECO with respect to the control specimen.

Autogenous shrinkages of all mixtures grow slowly on the first day. It is considered that the specimen may also swell with the hydration of the binder, and this part of swelling may cover part of the shrinkage on the first day. A similar result is also reported by Jiang when measuring autogenous shrinkage by ECDS [43]. One reason for this phenomenon is considered as the re-absorption of the bleeding water [52]. Another possible reason could be found on the scale of the hydration products [52,53].

The growth of autogenous shrinkage becomes quicker after one day. The specimen with SRA shows the smallest shrinkage in the first two days. However, the specimen with ECO turns to have the smallest autogenous shrinkage from 61 h after pouring. On the 7th day after pouring, the autogenous shrinkage of specimen with ECO is 26.3% lower than that of the specimen with SRA. The beneficial effect of SRA on shrinkage is due primarily to the descending of the surface tension in pore water [20,21,37]. The application of ECO also leads to the descending of the surface tension and lowers the autogenous shrinkage of AASC, which will be mentioned in Section 3.6.2. Another possible reason for shrinkage reduction may be attributed to the delay of hydration caused by a covering insolubility film on the GGBFS particles [28]. Moreover, another possible reason reported by another researcher is that the addition of oil in concrete has the ability to repel liquid water, inhabiting water movement through capillarity [49], which may further decrease the autogenous shrinkage.

The theoretical basis for the reduction of shrinkage may be caused by the reaction between the component of fatty acid in ECO and alkaline substance. The chemical reaction between ECO and alkaline substance in AASC is presumed as Equation (1). This reaction is usually called saponification reaction, which has a similar mechanism to the manufacturing process of soap. It is considered that this reaction may decrease the shrinkage stress and the chemical productions may delay hydration. Further study to prove this chemical reaction is needed.

2(C_17_H_35_COO)_3_C_3_H_5_ + 3CA(OH)_2_ = 3(C_17_H_35_COO)_2_Ca + 2C_3_H_5_(OH)_3_ (1)

The specimen with cooking oil shows larger shrinkage than other agents until the 3rd day after pouring. After 3 days, the specimen with EA shows a larger shrinkage than utilizing other agents. The reduction of autogenous shrinkage by applying EA is mainly due to the formation of secondary ettringite [48]. The increased volume of ettringite may decrease the shrinkage, especially in the early age. However, this early age expansion is insufficient to compensate the subsequent long-term shrinkage in AASC.

In comparison with cooking oil, ECO shows a better shrinkage reducing effect due to the increased dispersion of cooking oil. After emulsification, the saponification reaction between well-dispersed oil and Ca(OH)_2_ becomes more sufficient, thus improving the shrinkage reducing effect.

High shrinkage of AASC is considered an obstacle to widespread adoption. According to experimental results, using ECO is considered an easy and effective method to mitigate the autogenous shrinkage of AASC which may help its wide application.

### 3.5. Carbonation Depth of the Concrete

**Figure 7:**
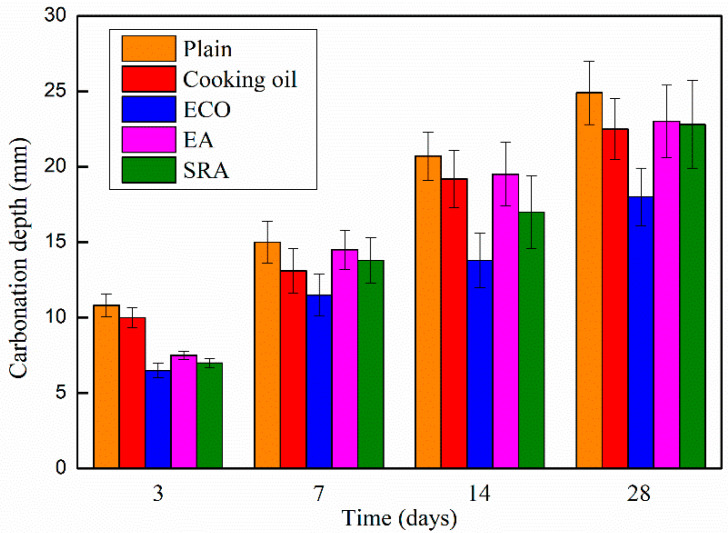
Carbonation depth of AASC with various of agents.

The carbonation depths of the AASCs with various agents are shown in Figure 7, which shows similar behaviors with other research [54]. With the addition of agents, carbonation depths of AASC specimens decrease at all periods in comparison with the control specimen, and the degree of the carbonation increases monotonically following age time. On the 3rd day, the cooking oil contained specimen shows a higher carbonation depth than specimens with other agents, but still lower than that of the control specimen. While the other agents, ECO, EA, and SRA, show similar contributions to the carbonation depth on the 3rd day. Among the four agents, ECO delivers the largest reduction on carbonation depth causing a smaller carbonation depth of 39.8% when compared with the plain specimen on the 3rd day. The gap of the carbonation depths between the plain and specimens with cooking oil, EA, and SRA becomes narrow in general following age time. On the 28th day, specimens with cooking oil, EA, and SRA reach an almost identical carbonation depth of approximately 10% lower than the control specimen. Utilizing SRA and EA slightly decreases the carbonation depth of AASC specimens. During the shrinkage process, AASCs are significantly shrunk leading to the development of micro-cracks that facilitate the ingress of CO_2_ into the concrete. SRA and EA may inhibit the formation of these micro-cracks because of the shrinkage-reducing and expansion effect [47,55]. Thus, the carbonation resistance of AASC is slightly improved by the addition of SRA and EA.

The largest reduction of carbonation depth (27.7%) is in AASC with ECO on the 28th day. The main reason for the decrease in carbonation depth is the lowering of porosity of the AASC caused by ECO, as illustrated by the MIP data in Section 3.6.1. The denser inner structure could mitigate the penetration of CO_2_ and decelerate the carbonation.

### 3.6. Mechanism of Shrinkage Reducing Effect of ECO

#### 3.6.1. MIP Analysis of AASP with ECO

Pore sizes are generally classified in accordance with the International Union of Pure and Applied Chemistry (IUPAC) system [56,57] and results are provided in Table 7. Both the mesopores and macropores belong to the capillary pores. Shrinkage of concrete highly depends on the volume of the mesopores [58].

The results of the MIP tests of AASP with 0%, 2%, and 4% ECO by the mass of GGBFS are provided in Table 8. With the addition of ECO, the intrusion volume and total pore area are decreased. The porosity of the paste with 2% ECO decreases from 27.99% to 23.21% indicating that the addition of ECO makes the structure denser than plain in AASC. Median and average pore diameters are also decreased by applying ECO. The addition of ECO results in finer pore sizes than plain. However increasing the dosage of ECO to 4% would not alter pore sizes significantly and all test results are only slightly decreased when compared with the dosage of 2% ECO.

**Figure 8:**
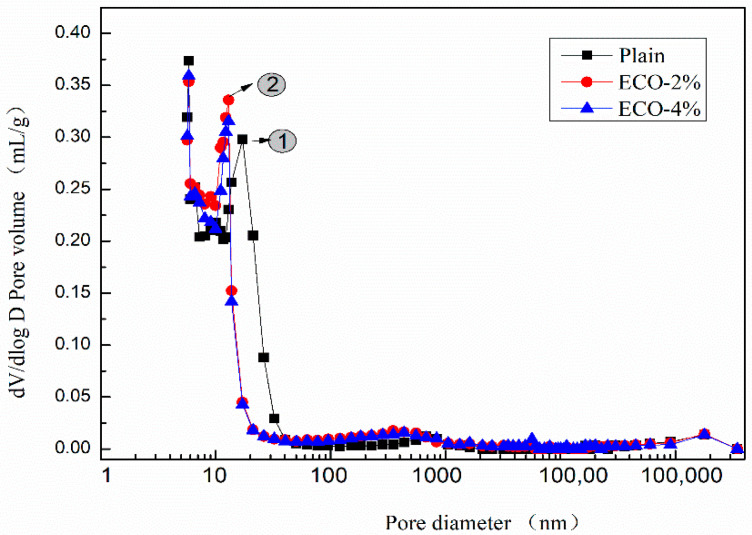
dV/dlog D pore size distribution.

Figure 8 shows the dV/dlog D pore size distribution curves of the plain and pastes with 2% and 4% ECO. Although mixtures with ECO show a slightly larger pore volume in the pore sizes range of 200–500 nm, patterns of pore volume distribution of the three mixtures are very similar when the pore sizes are larger than 40 nm or smaller than 13 nm. The addition of the ECO leads to a shift of the main peak to a smaller size. The peak of the pore size of the plain mixture appears around 17 nm (peak No.1), whereas it occurs around 13 nm in both ECO-2% and ECO-4% mixtures (peak No.2). The pore sizes in the range of 17 nm to 27 nm decrease about 90% with respect to the plain after the addition of ECO. These pores belong to the mesopores and the decreased volume of these pores may be considered as a reason for the reduction of autogenous shrinkage [59]. The pore properties of mixes with 2% and 4% ECOs are very similar. Results show the addition of ECO fills the pores from 14 nm to 40 nm and makes some of them change size to 11–13 nm. The total volume of pores decreases with the addition of ECO, but is insensitive to the dosage of ECO. Similar results are also reported by Liu [27], the addition of cooking oil in OPC-based concrete also leads to the refinement of pores and decreases porosity.

**Figure 9:**
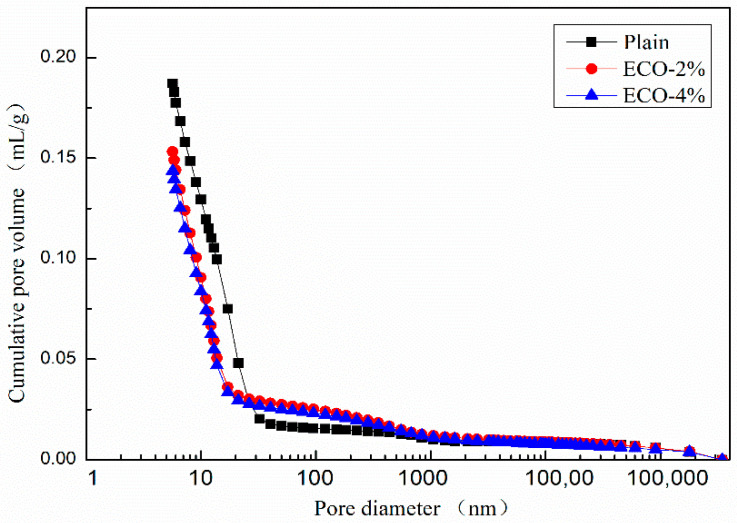
Cumulative pore size distribution.

Figure 9 shows the cumulative pore size distribution curve of the mixes. The variation of the cumulative pore volumes of mixes with ECO could be divided into three regions according to the pore size: Pores with a diameter smaller than 20 nm, in the range between 20 nm to 400 nm, and larger than 400 nm. The cumulative pore volumes of mixes with ECO are smaller than the corresponding plain in the first zone and become larger than the corresponding plain in the second zone. While in the third zone, the cumulative volumes of mixes with and without ECO are almost identical. Figure 9 also illustrates that the dosages of ECO have a very limited influence on cumulative pore volumes. These results indicate that the addition of ECO could increase the compactness of the inner structure with smaller pore sizes of AASC.

The pore size distribution may partially explain the positive influence of ECO on autogenous shrinkage and resistance of carbonation. As the shrinkage of concrete is highly dependent on the volume of mesopores, the addition of cooking oil decreases the volume of mesopores in AASC, thus resulting in the decrease of the autogenous shrinkage. The carbonation of concrete is generally known as a neutralizing process, CO_2_ from air intrudes into concrete and reacts with the alkali component. With the addition of ECO, the porosity of AASC is decreased and the intrusion of CO_2_ into the internal of AASC may become more difficult. The filled pores resist the intrusion of CO_2_ and decelerate the carbonation of AASC.

#### 3.6.2. Surface Tension of ECO Solution

The autogenous stress of concrete is directly proportional to the surface tension of pore solution [57,59]. Decreased surface tension may lead to the decline of autogenous shrinkage.

**Figure 10:**
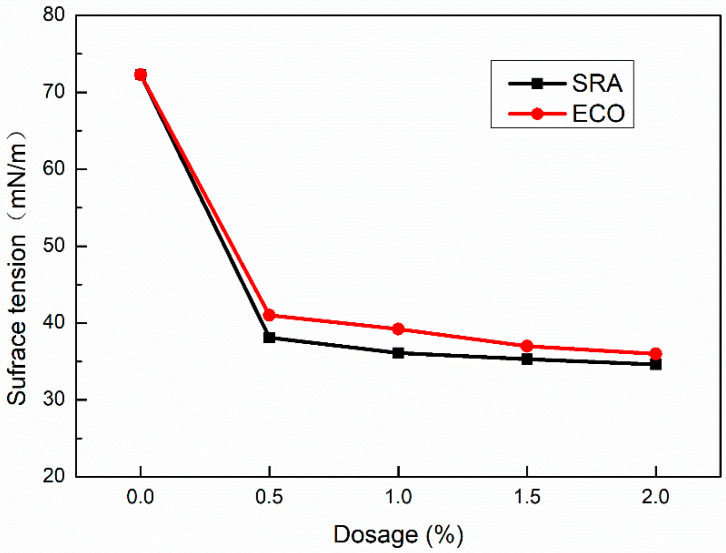
Surface tension of solution with various dosage of ECO.

Figure 10 shows the variation of the solution’s surface tension against dosages of ECO and SRA which was tested by the tensiometer at room temperature. The surface tension of pure water is 72.3 mN/m. With the addition of ECO, the surface tension decreases significantly. Following the addition of ECO from 0.5% to 2%, the surface tension reaches in the range of 41.0 mN/m to 36.0 mN/m varying in a narrow amplitude. Utilizing SRA shows a very slightly lower surface tension than using ECO. According to many researchers [20,29,34], the lower surface tension of concrete is one of the main reasons for ECO and SRA in AASC, which can significantly reduce shrinkage.

#### 3.6.3. SEM Analysis

**Figure 11:**
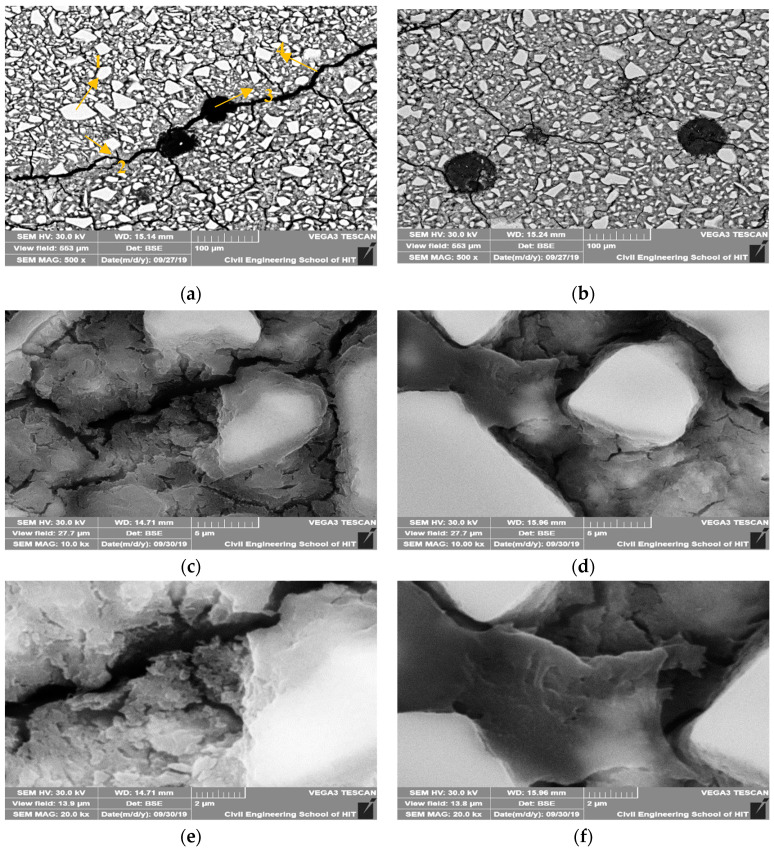
Backscattered electrons (BSE) images of AASC of plain (**a**) Control (Magnify 500 times) (1) Unreacted slag particle (2) Hydration products (3) Pore (4) Micro-cracks, (**b**) ECO-2% (Magnify 500 times) (**c**) Plain (Magnify 10,000 times), (**d**) ECO-2% (Magnify 10,000 times), (**e**) Plain (Magnify 20,000 times) and with 2% ECO, (**f**) ECO-2% (Magnify 20,000 times).

Figure 11 shows the BSE images for the plain AASP mixture and AASP mixture with 2% ECO after 28 days of curing with different magnifications.

With the addition of ECO, no matter the magnification times, micro-cracks of specimens were obviously decreased. The micro-cracks existed in the control mixture are generally wider than the corresponding micro-cracks in the mixture with ECO. It is considered that the addition of ECO could decrease the micro-cracks caused by autogenous shrinkage in the GGBFS based binder. Two reasons may explain this phenomenon. The first reason is the decrease of the surface tension caused by the ECO mentioned above. The second reason is that the addition of ECO in AASC may also lead to the saturation of the pores and decrease shrinkage.

Through the observation of the images magnified for 10,000 and 20,000 times, plain AASP mixture seems to have a more porous structure at the hydrated part of GGBFS than that of the ECO-containing mixture. It is considered that by applying ECO, the capillary pores are decreased and the hydrated products became denser. This result complies with the above MIP test results. The decrease of the capillary pores by applying ECO may also explain the reduction of autogenous shrinkage of AASC.

## 4. Conclusions

In this study, ECO was applied to AASC as a shrinkage-reducing agent and compared with SRA, EA, and cooking oil. The results indicated that cooking oil after emulsification could significantly reduce the autogenous shrinkage of AASC. The following conclusions could be drawn:

- The addition of cooking oil and ECO slightly decreased the flowability of concrete due to the increase of the viscosity. However, the influence of ECO on the flowability of concrete was weaker than that of cooking oil without emulsification. The utilization of SRA showed the best flowability in all the four types of agents;
- With the exception of using EA, setting times of concrete were prolonged with the addition of agents used. Particularly, the addition of ECO could sharply retard the initial setting time of AASC due to a covering insolubility film on the GGBFS particles which may delay hydration. Through the application of ECO, AASC showed an acceptable setting time which is considered to benefit the application of AASC in-situ;
- Compressive strength was substantially reduced at an early age with the addition of agents. However, the ECO would deliver higher compressive strength among the four agents. The decreased early age strength caused by ECO and cooking oil was attributed to the delay of the hydration. With age developed, the negative influence of ECO on the compressive strength was weakened. For the 28th day compressive strength, AASCs with and without ECO were almost the same;
- All agents could reduce the autogenous shrinkage of AASC. Among them, ECO showed to have a better effect on the reduction of shrinkage. Compared with the plain mixture, the utilization of ECO could reduce 66.8% of autogenous shrinkage on the 7th day due to the decrease of the surface tension and saponification reaction. This indicates that the application of ECO on AASC may provide a solution for the application of high autogenous shrinkage problem of AASC;
- The addition of ECO could enhance the carbonation resistance of AASC. The carbonation depth of AASC on the 28th day was reduced 27.7% by the use of ECO. The reduction of carbonation depth was mainly caused by the denser inner structure of AASC;
- Porosity and capillary pores volume of AASC decreased with the addition of ECO. Moreover, the surface tension of the AASC decreased with the addition of ECO, which are the origination of the reduction of autogenous shrinkage.

In comparison to SRA and EA, using ECO as a shrinkage-reducing agent showed better durability and mechanical performance. ECO could also deliver a setting retardant effect and higher carbonation resistance, which can be considered beneficial for applying AASC on a construction site.

AASC is a type of eco-friendly material and with a superior performance over OPC-based concrete in many aspects. The high shrinkage, rapid setting, and high carbonation depth of the AASC are considered the three main drawbacks that limit the application of AASC. In view of the results conducted by this paper, utilizing ECO may provide a solution for these drawbacks, and become beneficial to the wider application of AASC. Moreover, in comparison with other agents, cooking oil-based ECO is widely available around the world. Utilizing ECO is considered a cheap and easy way to overcome the limitations of AASC.

## Figures and Tables

**Table 1:** Chemical composition of Ground-granulated blast furnace slag (GGBFS) (wt.%).

	CaO	SiO_2_	Al_2_O_3_	MgO	SO_3_	TiO_2_	Na_2_O	Fe_2_O_3_	L.O.I
GGBFS	40.95	30.74	14.87	8.95	0.81	1.53	0.37	0.52	0.76

**Table 2:** Properties of fine and coarse aggregates.

Aggregates	Density (g/cm^3^)	Water Absorption (%)	Modulus Fineness
River Sand	2.88	1.40	2.87
Crushed Gravel	2.86	1.21	–

**Table 3:** Properties of agents.

Agents	Density (g/cm^3^)	Condition	Colour	Main Component
OP-10	0.98	Liquid	Yellow	C_35_H_64_O_11_
Span-80	0.92	Liquid	Transparent	Polyoxyethylene Sorbitan Fatty Acid Ester
Expansion agent (EA)	1.40	Solid	White	Sulphoaluminate, Gypsum
Shrinkage reducing agent (SRA)	0.96	Liquid	Faint Yellow	Polyether Derivative

**Table 4:** Components of the emulsified cooking oil (ECO) solution (wt.%).

Solution	Cooking Oil	Deionized Water	Span-80	OP-10
ECO Solution	57	40	2.7	0.3

**Table 5:** Concrete mixture proportion (kg/m^3^).

Constituents	Control	Cooking Oil		ECO		EA		SRA	
GGBFS	360	360		360		360		360	
Water Glass	53.89	53.89		53.89		53.89		53.89	
NaOH	12.83	12.83		12.83		12.83		12.83	
Water ※	180 (145.31)	180 (145.31)		180 (145.31)		180 (145.31)		180 (145.31)	
W/B	0.5	0.5		0.5		0.5		0.5	
Fine Aggregates	747.69	747.69		747.69		747.69		747.69	
Coarse Aggregates	1121.54	1121.54		1121.54		1121.54		1121.54	
Admixture	–	Cooking Oil	7.2	Cooking Oil	7.2	EA	7.2	SRA	7.2
				Span-80	0.486				
				OP-10	0.054				

※ Number in blanket means the quantity of free water without water contained in water glass. Density of raw materials. GGBFS: 2.94 g/cm^3^, water glass: 1.35 g/cm^3^, NaOH: 2.13 g/cm^3^, fine aggregate: 2.88 g/cm^3^, and coarse aggregate: 2.86 g/cm^3^.

**Table 6:** Setting time of alkali-activated slag paste with different agents (minutes).

Setting Time	Control	EA	SRA	Cooking Oil	ECO
Initial Setting	46	36	76	59	96
Final Setting	123	73	142	133	149

**Table 7:** International Union of Pure and Applied Chemistry (IUPAC) pore size classification.

Pore Description	Radius (nm)	Diameters (nm)
Micropores	<1.25	<2.5
Mesopores	1.25–25	2.5–50
Macropores	25–5000	50–10,000
Entrained Air Voids, Entrapped Air Voids, Pre-Existing Microcracks	5000–50,000	10,000–100,000

**Table 8:** Results of mercury intrusion porosimetry (MIP) tests.

Test Items	Plain	ECO-2%	ECO-4%
Total Intrusion Volume (mL/g)	0.1872	0.1532	0.1437
Total Pore Area (m^2^/g)	59.113	53.299	50.559
Median Pore Diameter (Volume, nm)	14.5	11.4	11.3
Median Pore Diameter (Area, nm)	9.4	8.4	8.3
Average Pore Diameter (4V/A, nm)	12.7	11.5	11.4
Porosity (%)	27.9882	23.2084	21.9045
